# Contrasting Embryogenic Trajectories Reveal Spatial Control as a Key Determinant of Embryogenic Competence in *Theobroma cacao* L.

**DOI:** 10.1111/ppl.71072

**Published:** 2026-08-26

**Authors:** Stefhania Alzate‐Lozano, Inaê Mariê de Araújo Silva Cardoso, Claudia Franca Barros, Uilson Vanderlei Lopes, Jonny Everson Scherwinski‐Pereira

**Affiliations:** ^1^ Department of Forest Engineering University of Brasília (UnB) Brasília Brazil; ^2^ MAPA/CNPq Embrapa Postoctoral Fellowship Program Brasília Distrito Federal Brazil; ^3^ Rio de Janeiro Botanical Garden National School of Tropical Botany Rio de Janeiro Rio de Janeiro Brazil; ^4^ Cocoa Research Center (CEPEC) Executive Commission of the Cocoa Farming Plan (CEPLAC) Ilhéus Bahia Brazil; ^5^ Embrapa Genetic Resources and Biotechnology Brasília Distrito Federal Brazil

**Keywords:** auxin, embryogenic competence, non‐regenerative response, procambium, recalcitrance, somatic embryogenesis

## Abstract

Somatic embryogenesis (SE) in *Theobroma cacao* is a micropropagation technique of great importance but remains constrained by genotype recalcitrance. The cellular and molecular mechanisms distinguishing regenerative from non‐regenerative responses remain poorly understood. Here, we elucidate the ontogenetic events governing embryogenic competence by comparing responsive genotypes (CCN‐51 and 752) with a recalcitrant one (PH16). Our results demonstrate that SE success in cacao is not determined by cell proliferation speed, but rather by a slow, organized, and spatially confined developmental trajectory. Through histological and immunofluorescence analyses, we show that responsive genotypes establish a focused response within procambial tissues. This leads to the formation of late, localized auxin maxima (after 42 days), which are essential for organizing morphogenesis. In contrast, the recalcitrant genotype exhibits a rapid, diffuse, and disorganized response that fails to establish necessary hormonal gradients, resulting in developmental collapse. We conclude that embryogenic competence in cacao should be redefined: it is not merely an induction event, but the precise orchestration of a developmental program dependent on the controlled reactivation of a vascular stem cell niche. These findings provide novel morphological and hormonal markers for selecting elite genotypes and offer new strategies to overcome recalcitrance in cacao.

## 1 Introduction

Somatic embryogenesis (SE) is a powerful tool for regenerating whole plants from somatic cells, harnessing cellular plasticity beyond the zygote (Nic‐Can et al. 2015; Fehér 2019; Hazubska‐Przybył et al. 2020; Long et al. 2022). This process involves the reprogramming of normal cell fate, allowing somatic cells to acquire totipotency and originate new embryos (Hazubska‐Przybył et al. 2020; Su et al. 2021; Fehér 2023). Under in vitro conditions, SE can be induced from various plant explants, although not all cells possess embryogenic potential (Fehér 2019, 2023), which compromises protocol efficiency.

Among the main stimuli for SE induction, plant growth regulators, particularly auxins and cytokinins, stand out, as they not only promote cell division but also act as molecular signals capable of modulating the expression of key genes (Pasternak et al. 2002; Bishopp et al. 2011; Hazubska‐Przybył et al. 2020; Zhang et al. 2021; Karami et al. 2023). Auxins are considered the main triggers of this cellular reprogramming. Synthetic auxins such as 2,4‐dichlorophenoxyacetic acid (2,4‐D) tend to be more effective than indole‐3‐acetic acid (IAA) due to their low affinity for PIN‐FORMED (PIN) efflux carriers, favoring intracellular accumulation and activation of the embryogenic response (Delbarre et al. 1996; Šimášková et al. 2015; Hu et al. 2021; Balcerowicz 2024; Karami et al. 2024). These data indicate that not only the presence but also the precise spatial control of auxin distribution is critical for successful SE. Conversely, excessive, or prolonged auxin accumulation may lead to developmental abnormalities, highlighting the complexity of hormonal homeostasis (Hu et al. 2021; Balcerowicz 2024; Karami et al. 2024).

Understanding the spatial and temporal patterns of hormonal accumulation during SE is essential for improving cloning protocols, particularly in commercially valuable crops such as cacao ( *Theobroma cacao* L.; Henao‐Ramírez et al. 2018; Guillou et al. 2018; Daouda et al. 2019; Guillou and Verdier 2022; Henao Ramirez et al. 2023; Bustami and Werbrouck 2024). Despite its potential, in vitro culture of cacao remains limited by strong genotypic dependence and the recalcitrance of some elite genotypes (Maximova et al. 2002; Guillou et al. 2018; Daouda et al. 2019; Henao Ramirez et al. 2023; Bustami and Werbrouck 2024). Although significant advances have been made in understanding the general process, the factors determining why certain genotypes establish proper anatomical and hormonal patterns while others fail remain unclear in cacao. In particular, the early cellular events involved in the acquisition of embryogenic competence are still poorly characterized, especially during the initial stages of induction.

In this context, the present study aimed to characterize and compare the ontogeny of SE in three contrasting *T. cacao* genotypes. Through histological, histochemical, and immunofluorescence analyses for auxin localization, we sought to identify cellular events and hormonal patterns capable of distinguishing efficient regenerative trajectories from non‐regenerative pathways. Elucidating these factors is fundamental for optimizing clonal propagation protocols and enabling more effective strategies to overcome recalcitrance in elite genotypes.

## 2 Materials and Methods

### 2.1 Plant Material

This study was conducted with three contrasting genotypes of *T. cacao* obtained from the collection of Embrapa Genetic Resources and Biotechnology, Brasília, DF, Brazil, and cultivated in a nursery from mother plants maintained at the Experimental Field of the Executive Commission of the Cocoa Farming Plan (CEPLAC), in Ilhéus, BA, Brazil. Genotypes 752 and CCN‐51 were previously characterized as responsive to somatic embryogenesis (RE; Oliveira et al. 2025), whereas genotype PH16 was considered non‐responsive or recalcitrant (NRE). CCN‐51 is a clone developed in Ecuador and widely planted in many countries; PH16 is a Brazilian clone also widely planted, whereas accession 752 represents a wild cacao genotype used as an embryogenic responsiveness control in our laboratory.

Staminodes were excised from closed floral buds measuring 4–5 mm in length and used as explants. Disinfestation was performed by immersion in 70% (v/v) ethanol for 3 min, followed by treatment with sodium hypochlorite (NaOCl) containing 0.5% active chlorine for 10 min, and three consecutive rinses with autoclaved distilled water, each lasting 5 min.

Five staminodes from each bud were inoculated in Petri dishes (15 × 90 mm) containing 25 mL of somatic embryogenesis induction medium according to Li et al. (1998). The protocol involved three phases: (i) 2 weeks in primary callus medium (PCM, DKW salts; Driver and Kuniyuki 1984); (ii) 2 weeks in secondary callus medium (SCM, WPM salts; Lloyd and McCown 1980); and (iii) 4 weeks in embryo development medium (EDM, also based on WPM salts). All media were supplemented with 2.5 gL^−1^ Phytagel, adjusted to pH 5.8 ± 0.1, and sterilized by autoclaving at 121°C for 20 min under 1.3 atm pressure. Cultures were maintained in darkness at 25°C ± 2°C and 60% relative humidity.

### 2.2 Anatomical and Histochemical Analyses

To characterize the ontogeny of SE, samples were collected at eight distinct time points: 0, 3, 7, 14, 21, 28, 42, and 56 days after induction. At each time point, five staminodes per genotype were analyzed, totaling 120 explants (five staminodes × three genotypes × eight time points). The experiment was repeated twice independently. Samples included explants with and without callus formation, calli of different morphologies, and somatic embryos. After fixation, representative histological sections were obtained, covering various explant regions.

Fixation was performed in a modified Karnovsky solution composed of 4% paraformaldehyde, 2.5% glutaraldehyde, and 0.05 M sodium cacodylate buffer (pH 7.2) for 24 h, with the first hour under vacuum. Samples were then washed three times in cacodylate buffer and dehydrated through a graded ethanol series (30%, 50%, 70%, and twice in 100%), each step lasting 1 h under vacuum. Tissues were then embedded in historesin (Leica) according to the manufacturer's instructions. Longitudinal and transverse sections of 3–7 μm thickness were obtained using a rotary microtome (Leica RM2125 RTS) and mounted on slides preheated to approximately 40°C.

**FIGURE 1:**
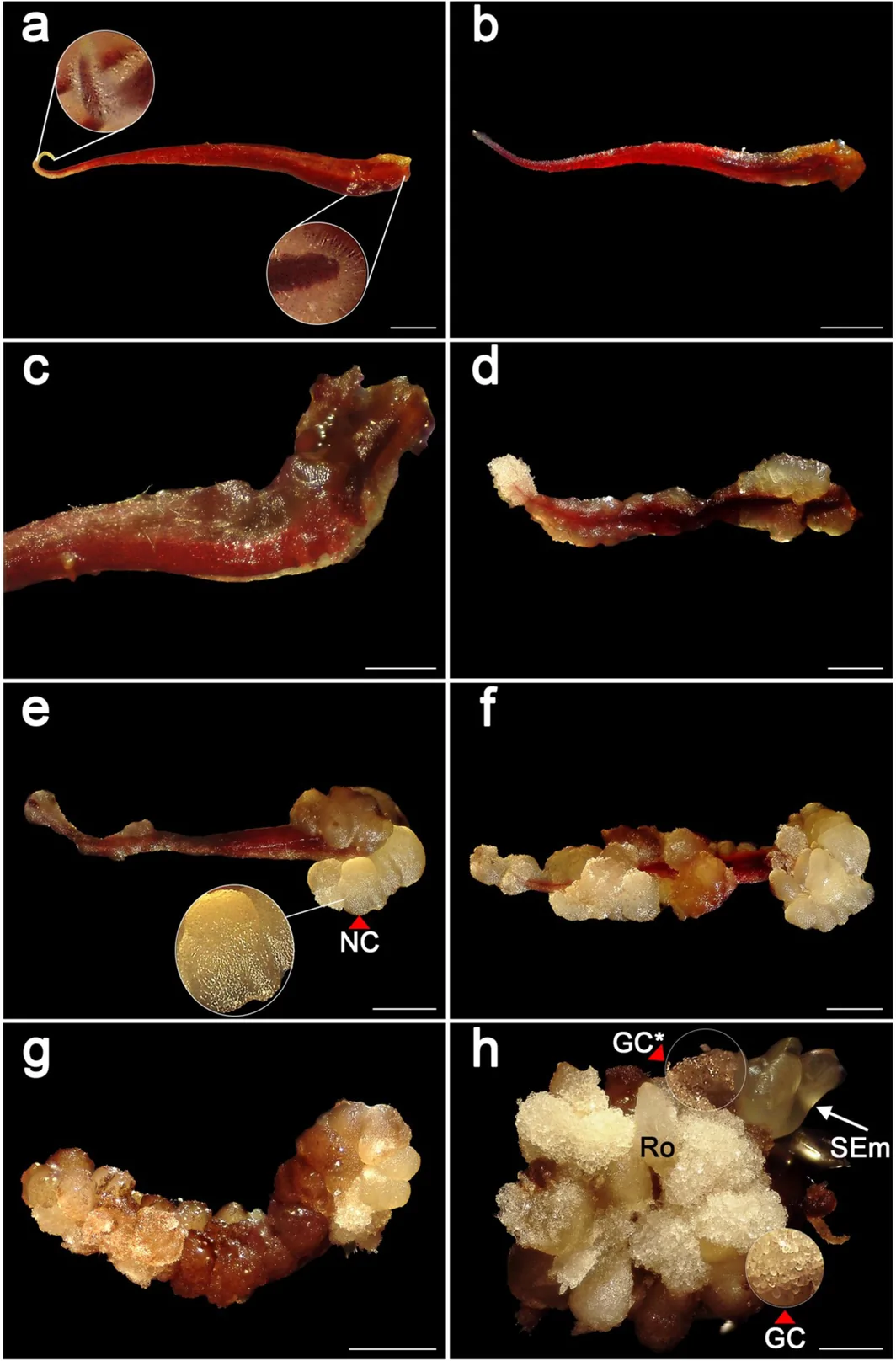
External morphology of *Theobroma cacao* staminodes during different stages of somatic embryogenesis. (a) Fresh staminode at day 0, showing intense wine coloration, clear distal extremity, and presence of trichomes. (b) Staminode after culture in callus induction medium for 3 days, with initial swelling in the basal region. (c–h) Subsequent stages of somatic embryogenesis, showing callus development and formation of somatic embryos; GC*, embryogenic granular callus; GC, granular callus; NC, nodular callus; Ro, root; SEm, somatic embryos. Symbol: White arrow indicates the presence of somatic embryos. (c): 7 days; (d): 14 days; (e): 21 days; (f): 28 days; (g): 42 days; (h): 56 days. Scale bars: (a, c): 0.5 mm; (b, d, e, f, h): 1 mm; (g): 2 mm.

Histological sections were stained with 0.5% toluidine blue (O'Brien et al. 1965) and analyzed under a Leica DM 750 light microscope using the Leica Application Suite EZ software. Based on morphoanatomical evaluation, calli were classified into three distinct morphological categories according to Oliveira et al. (2025): Non‐embryogenic granular callus: Irregular surface, loosely organized cells, low‐density cytoplasm, and large vacuoles indicating low metabolic activity and absence of meristematic organization (Figures 1h and 4k). This callus type typically exhibited a whitish tone. Embryogenic granular callus: Densely cytoplasmic cells with prominent nuclei and nucleoli, and small vacuoles. These cells, irregularly distributed on the callus surface, often formed clusters of embryogenic cells and structures resembling proembryos at the edges, exhibiting variable brownish coloration ranging from light to dark shades (Figures 1h and 4n). Embryogenic nodular callus: Compact structure with well‐defined meristematic centers composed of small, dense, isodiametric cells showing intense mitotic activity (Figures 1e and 4k,o).

Additionally, sections were subjected to histochemical staining with aniline blue to detect callose (α‐1,3‐glucans), following the method of Eschrich and Currier (1964). Observations were made under fluorescence microscopy with UV excitation light (340–380 nm). Cell diameters were measured using the ImageJ software (Rasband, W.S., ImageJ, U.S. National Institutes of Health).

### 2.3 Confocal Microscopy

IAA immunolocalization analyses were performed in staminode‐derived cultures of the responsive genotype 752 collected at 0, 3, 7, 14, 21, 28, 42, and 56 days after induction. Samples used in the confocal microscopy were fixed in a solution containing 4% paraformaldehyde, 2.5% glutaraldehyde, and 0.1 M phosphate‐buffered saline (PBS; pH 7.2) for 24 h. They were then washed three times in PBS (20 min each) and dehydrated through a graded ethanol series (10% to 100%), with three washes per concentration. After dehydration, tissues were gradually embedded in butyl methacrylate (BMM, Sigma) through a controlled transition from ethanol to pure BMM. Polymerization was carried out under UV light for 7 days at −20°C. Sections of 4–6 μm thickness were obtained using a rotary microtome and mounted on slides treated with 3‐aminopropyltriethoxysilane (ImmunoSlide, EasyPath), then dried at 42°C.

### 2.4 Immunolabeling

Endogenous auxin detection was performed by immunolabeling using a monoclonal anti‐indole‐3‐acetic acid antibody (anti‐IAA; Sigma, A0855), following the protocol adapted from Corredoira et al. (2017). Sections were first washed three times in PBS (3 min each) and then subjected to nonspecific blocking with 5% BSA in PBS for 10 min. Subsequently, they were incubated with the primary antibody diluted 1:100 in 1% BSA.

After another series of PBS washes, slides were incubated in the dark for 45 min with the secondary antibody Alexa Fluor 594 goat anti‐mouse IgG (H + L; Thermo Fisher) diluted 1:200 in PBS, according to Testillano et al. (2013). Slides were then washed again in PBS, mounted with Fluoromount‐G mounting medium (Thermo Fisher E140370), and analyzed under a laser scanning confocal microscope (Leica TCS SPE; Leica Microsystems) with an excitation laser at 488 nm. Acquisition parameters were standardized across all samples. For each sampling time, three independent staminodes (biological replicates) were processed. From each staminode, two microscope slides (technical replicates) were prepared, and multiple sections were examined per slide. Representative sections showing consistent immunolabeling patterns among the biological replicates were photographed using identical microscope acquisition settings. Negative controls were prepared by omitting the primary antibody and replacing it with PBS, while maintaining all other steps of the procedure (El‐Tantawy et al. 2014).

## 3 Results

### 3.1 Morphoanatomical and Histochemical Description of the Ontogeny of Somatic Embryogenesis in *T. cacao*

The staminodes of *T. cacao* exhibited an intense wine‐red coloration and an average length of approximately 6 mm, being covered by long unicellular trichomes (Figure 1a). The distal end was pointed, with colors ranging from translucent white to yellow, while the base, emerging from the floral receptacle, was white and gradually acquired reddish tones along roughly one quarter of its total length (Figure 1a,b). Callus formation began at the basal region of the staminode (Figure 1c,d), progressively expanding until covering the entire surface of the tissue (Figure 1e–g), and ultimately culminating in the differentiation of somatic embryos (Figure 1h).

**FIGURE 2:**
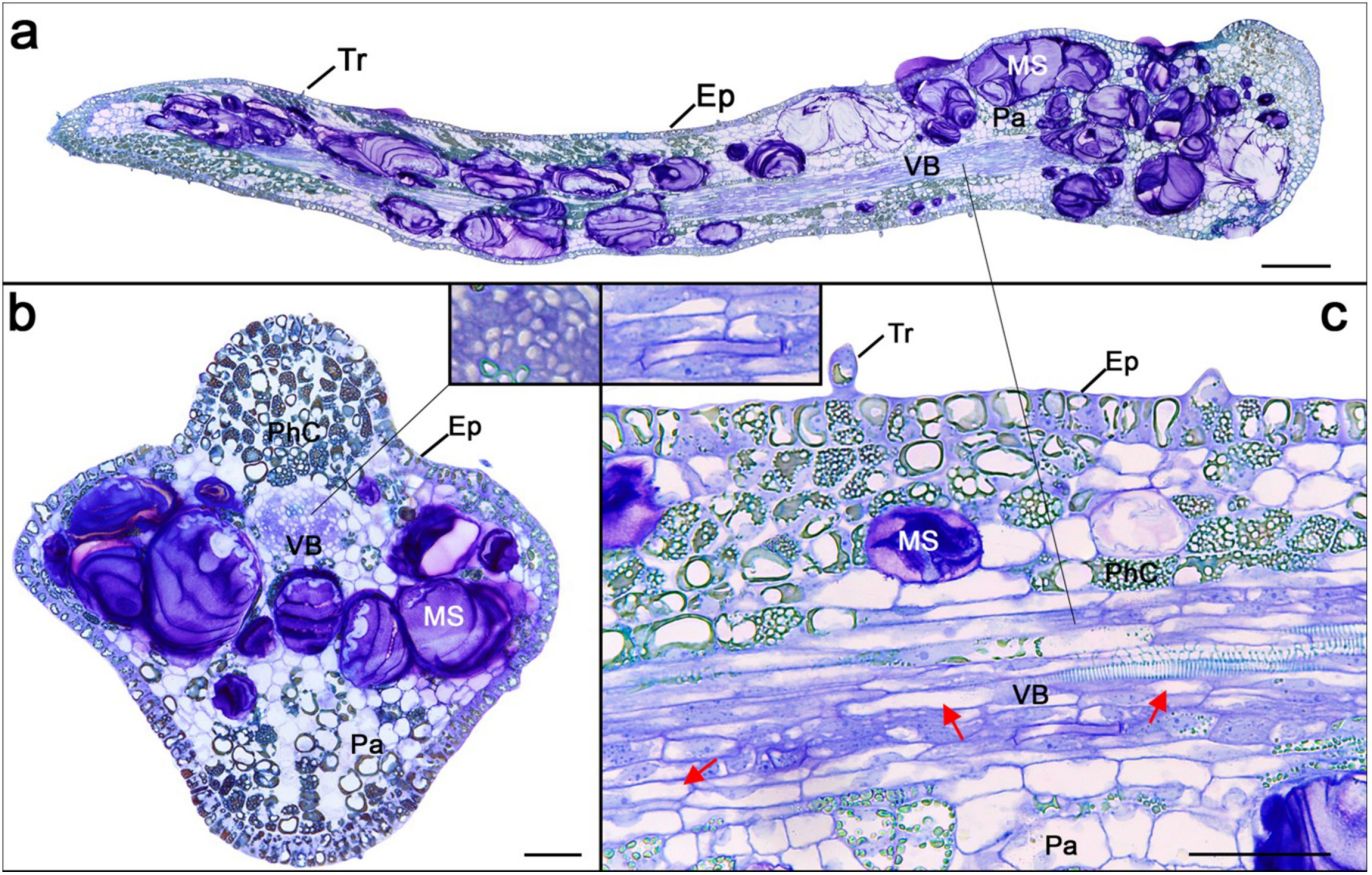
Histology of *Theobroma cacao* staminodes (0 day of culture). (a) Longitudinal section of the staminode. (b) Transverse section of the staminode, showing mucilage cells and central vascular bundle. (c) Detail of the epidermal, parenchyma, and vascular bundle cells. Ep, epidermis; MS, mucilage sac; Pa, parenchyma; PhC, phenolic compounds; Tr, trichome; VB, vascular bundle. Symbols: Red arrows are vacuoles. Scale bars: (a): 200 μm; (b, c): 50 μm.

Histological analysis at the initial time point (0 day), prior to somatic embryogenesis induction, revealed organized cellular structures. The uniseriate epidermis consisted of large vacuolated cells with peripheral cytoplasm and nuclei, as well as the presence of phenolic compounds (Figure 2a–c). The underlying parenchyma was mainly composed of vacuolated cells, some of which were rich in phenolic substances (Figure 2b,c). Large mucilage cavities were observed within the parenchyma, surrounded by vacuolated cells. The vascular bundle consisted of xylem with helical and annular thickenings, and phloem whose companion cells contained prominent nuclei (Figure 2b,c). The anatomical characteristics were consistent among all genotypes evaluated.

### 3.2 Early Events of Somatic Embryogenesis at Three and 7 Days

**FIGURE 3:**
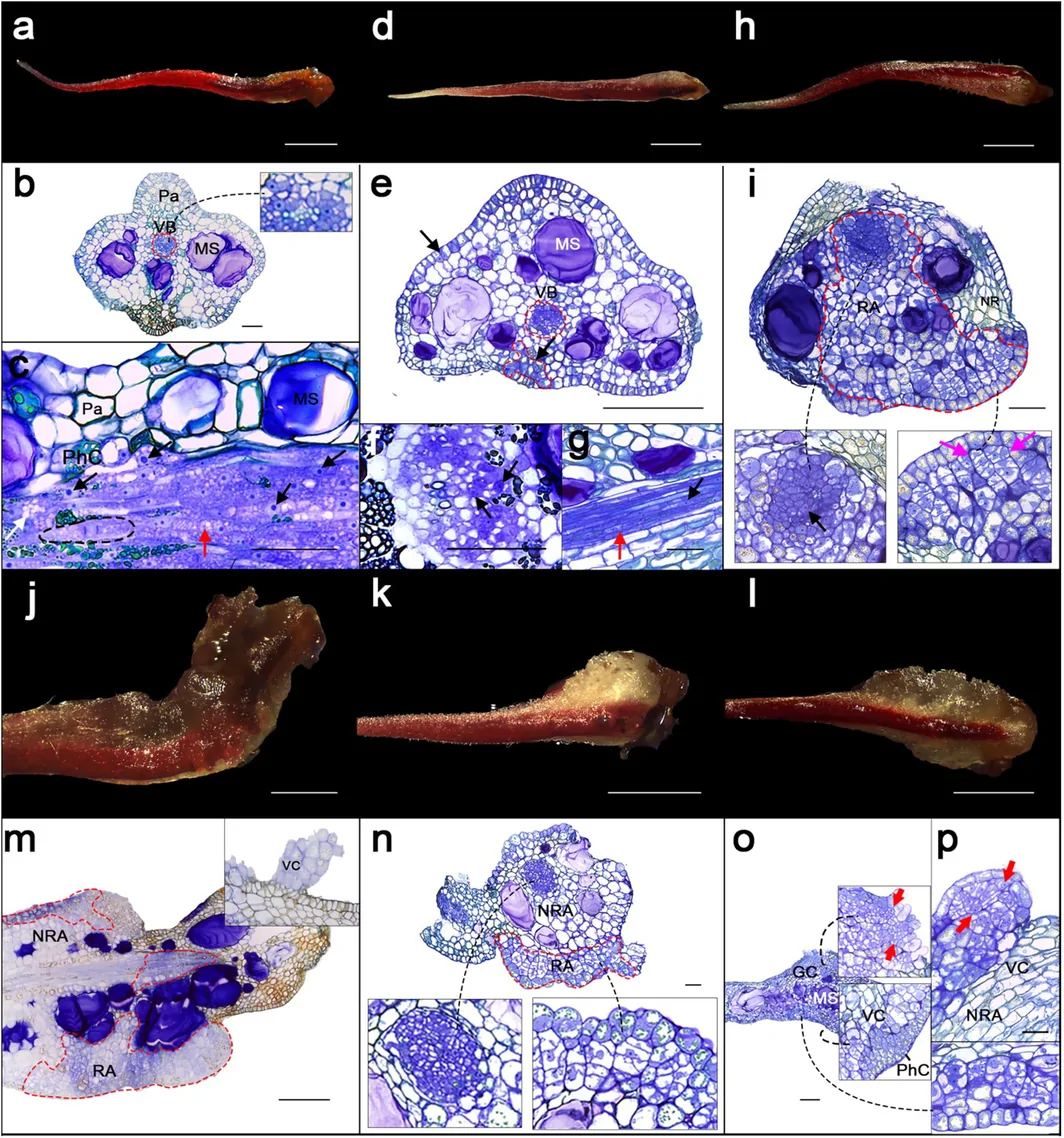
Histological responses of *Theobroma cacao* staminodes during the early stages of somatic embryogenesis induction. Responsive genotypes 752 (Column 1) and CCN‐51 (Column 2), and the recalcitrant genotype PH16 (Column 3), after three (a–i) and 7 days of culture (j–p). (a) Staminode after 3 days of culture. (b) Transverse section of the staminode showing a reactive zone restricted to the vascular bundle. (c) Detail of vascular bundle cells responding to the inductive stimulus, with visible cell divisions. (d) Staminode after 3 days of culture. (e) Transverse section of the staminode showing reactive zones in the vascular bundle and in epidermal and subepidermal cells. (f, g) Details of the vascular bundle with cells undergoing activation. (h) Staminode after 3 days of culture. (i) Transverse section of the staminode showing reactive zones (vascular bundle and parenchyma cells). (j, k, l) Staminodes after 7 days of culture. (m) Longitudinal section of a staminode after 7 days of culture, with red dotted lines delimiting anatomically reactive zones (RA); note signs of cell division in the vascular bundle and subepidermal region and the formation of protrusions with vacuolated cells (lateral detail). (n) Transverse section of the staminode showing reactive zones in the vascular bundle, epidermis, subepidermis, and parenchyma. (o) Longitudinal section of the staminode showing the onset of granular callus formation and reactive zones in the subepidermis. (p) Staminode protrusion with embryogenic cells. GC, granular callus; MS, mucilage sac; NRA, anatomically non‐reactive zone; Pa, parenchyma; PhC, phenolic compounds; RA, anatomically reactive zone; VB, vascular bundle; VC, vacuolated cell. Symbols: Black dotted lines are dividing cells; red dotted line represents the anatomically reactive zone; black arrows indicate cells with evident nucleus and nucleolus; pink arrows are periclinally dividing cells; red arrows are fragmented vacuoles. Scale bars: (a, d, h): 1 mm; (b, c, f, g, i, n, p): 50 μm; (j, k, l): 2 mm; (e, m, o): 200 μm.

After 3 days in primary callus induction medium, the staminodes exhibited initial morphological alterations, macroscopically visible as slight swelling (Figure 3a,d,h). The first histological changes indicated that the response originated in the vascular bundles of all genotypes (Figure 3b,c,e,f,i). Cells with embryogenic features displayed dense cytoplasm, prominent nuclei, and fragmented vacuoles (Figure 3c,f,g,i). The main distinction among genotypes was the spatial distribution of this initial response. In the responsive genotypes 752 and CCN‐51, cellular activity was concentrated mainly in the vascular tissues and in isolated epidermal or subepidermal regions (Figure 3b,c,e–g), whereas the recalcitrant genotype PH16 exhibited an early and diffuse proliferation, with a greater number of reactive cells distributed across multiple subepidermal layers (Figure 3i). At this stage, anatomically reactive (RA) and non‐reactive (NRA) areas were already distinguishable in PH16, showing broader and more prominent reactive regions. In contrast, in the responsive genotype CCN‐51, only a small active zone could be observed, as indicated by the dotted line (Figure 3e), whereas such distinction was not yet evident in 752.

After 7 days of culture in PCM medium, rounded and translucent protrusions appeared on the surface of the staminodes, mainly in the basal region, associated with tissue hyperhydration (Figure 3j–l). Histological analyses indicated that these structures originated from cell divisions in the epidermal and subepidermal layers, leading to surface rupture and the emergence of individual cells or small cell clusters (Figure 3m, upper corner detail). The outermost cells of these clusters contained large vacuoles responsible for the macroscopically visible protrusions. Multiple divisions were observed in the subepidermal region, occurring in different planes (Figure 3n). These divisions, oriented toward the epidermis, were associated with tissue swelling and hyperhydration (Figure 3o,p). At this point, RA and NRA zones also became clearly distinguishable in the responsive genotypes 752 and CCN‐51 (Figure 3m,n).

The accelerated and diffuse proliferation of the recalcitrant genotype PH16 resulted in the formation of larger calli (Figure 3l,o), suggesting a distinct developmental trajectory from the earliest stages of induction. Phenolic compounds were detected in both epidermal and subepidermal layers, in cells with and without embryogenic characteristics (Figure 3o,p).

### 3.3 Development of Embryogenic Calli and Induction of Somatic Embryos at 14 and 21 Days

**FIGURE 4:**
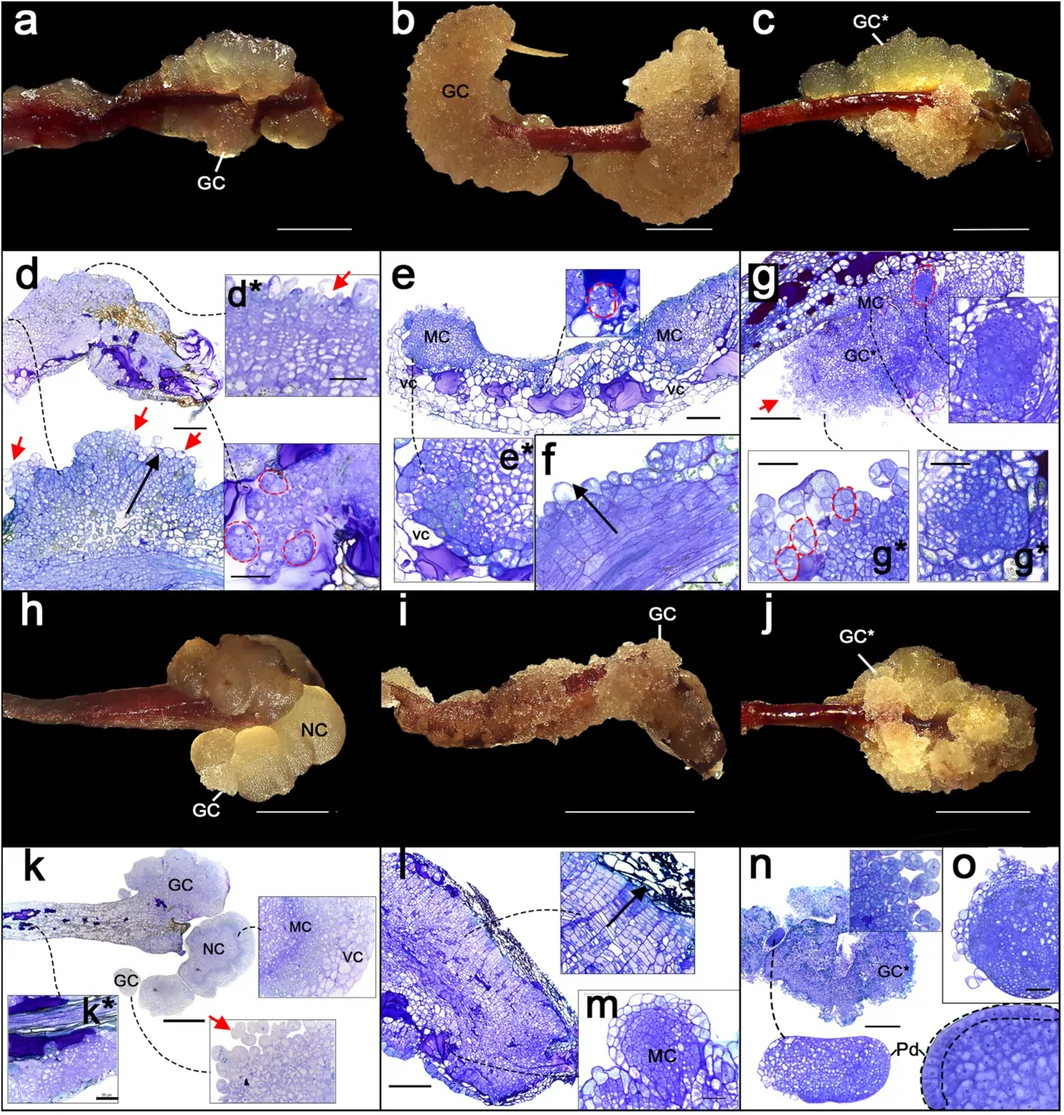
Histological changes in *Theobroma cacao* staminodes during the early stages of somatic embryogenesis induction. Responsive genotypes 752 (Column 1) and CCN‐51 (Column 2), and the recalcitrant genotype PH16 (Column 3) after 14 (a–g) and 21 (h–o) days of culture. (a–c) Morphology of staminodes after 14 days of culture. (d) Longitudinal section showing granular callus with periclinal cell divisions (black arrow) and the presence of proembryos (red dotted line) near mucilage sacs. (e) Longitudinal section highlighting meristematic centers and proembryos. (f) Detail of vascular bundle cells undergoing periclinal division (in the direction of the arrow). (g) Longitudinal section with granular callus showing meristematic centers and proembryos located at the edge and inner region of the callus. (h–j) Morphology of staminodes after 21 days of culture. (k) Longitudinal section showing granular and nodular calli (black arrow). (l) Tissue thickening caused by periclinal divisions. (m) Callus with a layer of vacuolated cells and clusters of meristematic cells. (n) Longitudinal section of a granular callus containing a structure consistent with an early‐stage somatic embryo, with an organized protoderm surrounding central cells. (o) Nodular callus composed of cells with dense cytoplasm. GC*, embryogenic granular callus; GC, granular callus; MC, meristematic center; NC, nodular callus; Pd, protoderm; VC, vacuolated cell. Symbols: Red dotted line represents proembryos; black arrows indicate periclinal divisions; red arrows indicate vacuolated cells. Images marked with * (d*, g*, k*) correspond to enlarged details of the main images (d, g, k, respectively). Scale bars: (a, c): 2 mm; b: 1 mm; (d, e, g, l): 200 μm; (d*, f, g*, k*, m, o): 50 μm; k, n: 500 μm.

After 14 days of culture, all genotypes exhibited granular calli resulting from intense proliferative activity (Figure 4a–c). However, the internal organization of these calli revealed distinct progressions along the embryogenic pathway among the responsive genotypes. Genotype 752 displayed the first proembryos (< 50 μm), usually located near mucilage sacs (Figure 4d, red dotted line), whereas CCN‐51 showed a more advanced stage, with well‐defined meristematic centers coexisting with proembryos (Figure 4e). Interestingly, the recalcitrant genotype PH16 also produced larger proembryos (> 50 μm) and meristematic centers. However, their broad and poorly segregated distribution, both within and at the periphery of the calli (Figure 4g), suggests a spatially less regulated proliferation compared with the responsive genotypes.

At 21 days, the transition to more organized stages was marked by the appearance of nodular calli (Figure 4h–j), a key milestone in the embryogenic pathway. These structures, originating from anatomically responsive regions, exhibited dense meristematic centers surrounded by parenchymatic cells and phenolic compounds (Figure 4k). In CCN‐51, a thickening of the tissue was evident, resulting from well‐organized periclinal cell divisions (Figure 4l), and the nodules contained highly organized meristematic clusters, indicating a stable progression of embryogenesis (Figure 4m).

In parallel, PH16 exhibited a paradoxical development. It formed nodular calli and even advanced structures resembling early somatic embryos, with a well‐organized protoderm (Figure 4n,o). Despite their morphologically promising appearance, these structures represented the peak of a developmental effort that was not sustained, failing to progress to mature embryos in later stages, in direct contrast with the trajectory of the responsive genotypes.

### 3.4 Induction of Somatic Embryos at 28 and 42 Days

**FIGURE 5:**
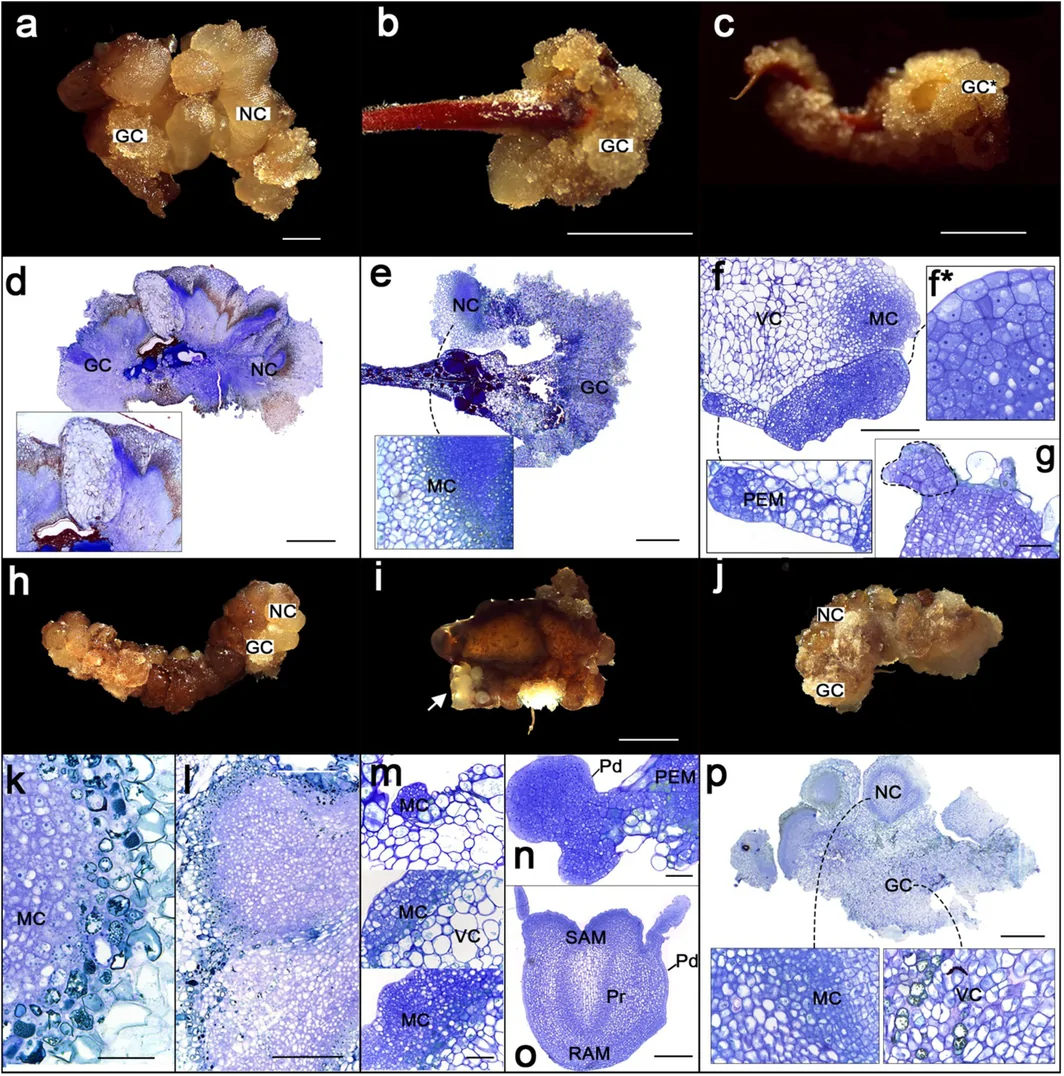
Progress of somatic embryogenesis in *Theobroma cacao* staminodes after 28 (a–g) and 42 (h–q) days of culture. Responsive genotypes 752 (Column 1) and CCN‐51 (Column 2), and recalcitrant genotype PH16 (column 3). (a–c) Morphology of staminodes after 28 days of culture. (d) Staminode with calli showing regions composed of vacuolated cells and areas containing poorly organized meristematic centers. (e) Staminode showing calli with regions of vacuolated cells interspersed with well‐defined meristematic centers. (f) Callus with expanding meristematic centers, vacuolated cells, partially connected clusters. (f*) Enlarged detail of a somatic embryo‐like structure. (g) Peripheral regions of the callus with proembryos. (h–j) Morphology of staminodes after 42 days of culture. (k) Nodular callus with defined meristematic centers. (l) Callus with actively dividing embryogenic cells. (m) Epidermal cells undergoing division and forming meristematic centers. (n) Somatic embryo and embryogenic callus. (o) Heart‐shaped somatic embryo showing the onset of cotyledon development. (p) Staminode of genotype PH16 at 42 days, showing nodular and granular calli predominantly non‐embryogenic. GC*, embryogenic granular callus; GC, granular callus; MC, meristematic center; NC, nodular callus; Pr, procambium; Pd, protoderm; PEM, pro‐embryogenic mass; RAM, root apical meristem; SAM, shoot apical meristem; VC, vacuolated cell. Symbols: Black dotted line indicates a proembryo. Scale bars: (a): 0.5 mm; (b, c, j): 2 mm; (d, e, q): 500 μm; (f, l, p): 200 μm; (g, k, m, n, o): 50 μm; (h): 2 mm; (i): 1 mm.

After 28 days of culture, all genotypes exhibited a significant increase in callus volume as a result of continuous cell proliferation (Figure 5a–c). However, their developmental trajectories became increasingly divergent. In the responsive genotypes, calli displayed organized meristematic centers (Figure 5d,e), although less prominent than those of the recalcitrant genotype PH16 (Figure 5f). The latter maintained intense yet deregulated proliferation, characterized by the emergence of pro‐embryogenic masses (PEMs), compact cell clusters with dense cytoplasm and apparent fragmentation or isolation, and embryo‐like structures showing early signs of protoderm formation, together with new meristematic centers and proembryos at the edges of non‐embryogenic granular calli (Figure 5f,f*). Proembryos were also observed at the callus margins (Figure 5g).

At 42 days, the staminodes were completely covered by calli, showing granular and nodular regions within the same explant (Figure 5h–j). At this stage, the responsive genotypes effectively initiated somatic embryogenesis. Genotype 752 exhibited clear progression, with nodular calli densely populated by active embryogenic cells, indicating a similar stage of maturation (Figure 5k,l). Genotype CCN‐51 was remarkable for producing somatic embryos at multiple developmental stages (Figure 5i,o). Some embryos arose from the cleavage or segmentation of larger meristematic masses (Figure 5m,n), highlighting the high level of organization in this genotype. In contrast, PH16 stagnated. Despite the intense proliferation observed at earlier stages, its calli were predominantly nodular or non‐embryogenic granular, with no formation of somatic embryos (Figure 5p). This limitation confirms the inability of the recalcitrant genotype to progress beyond the meristematic phase toward complete embryogenesis.

Under the conditions tested here, cacao embryogenic competence appears to reflect a precisely orchestrated developmental program anchored in the controlled reactivation of a vascular stem‐cell niche, rather than a simple induction event. The resulting developmental markers can guide elite‐genotype selection and strategies to mitigate recalcitrance.

### 3.5 Induction of Somatic Embryos at 56 Days

**FIGURE 6:**
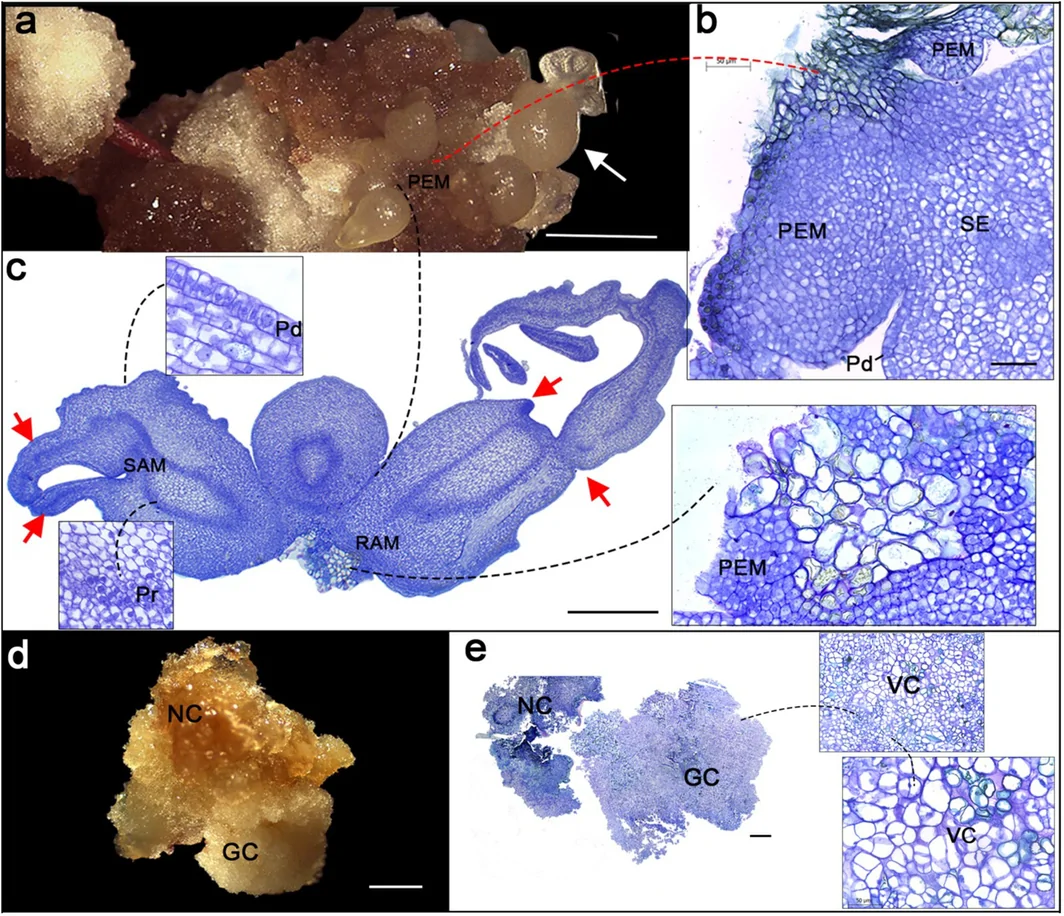
Advanced development of somatic embryogenesis in *Theobroma cacao* staminodes after 56 days of culture. (a) Staminode of the responsive genotype 752 completely covered by callus, showing somatic embryos (arrow). (b) Embryos partially connected to the callus, with new embryogenic masses organizing at the base, suggesting a multicellular origin. (c) Detail of a somatic embryo with protoderm (Pd), procambium (Pc), shoot apical meristem (SAM), and root apical meristem (RAM); embryogenic masses are visible at the base of the callus. (d) Callus of the recalcitrant genotype PH16, whitish in color, with no visible embryogenic structures. (e) Longitudinal section of a PH16 callus predominantly composed of highly vacuolated cells; cells with embryogenic morphology are restricted to small internal regions, with a reduced frequency of meristematic centers and proembryos compared to the responsive genotypes. GC, granular callus; NC, nodular callus; Pr, procambium; Pd, protoderm; PEM, pro‐embryogenic mass; RAM, root apical meristem; SAM, shoot apical meristem; SE, somatic embryo. Symbols: Red arrow indicate cotyledons; white arrow shows somatic embryos. Scale bars: (a): 2 mm; (b, e): 500 μm; (c): 50 μm; (d): 1 mm.

At 56 days of culture, the developmental trajectory of the responsive genotypes culminated in the formation of mature somatic embryos. In genotype 752, well‐developed embryos were observed at different stages (globular, heart‐shaped, and cotyledonary), with clearly defined tissues such as protoderm, procambium, and shoot and root apical meristems (Figure 6a–c). Demonstrating the robustness and recurrent nature of embryogenesis, new embryogenic cell masses (> 200 μm) continued to organize at the base of the calli, progressively isolating to give rise to new embryos (Figure 6b). The general morphology of these calli included nodular and granular regions with signs of intense mitotic activity. In more central areas, accumulations of phenolic compounds were detected and, occasionally, structures compatible with root formation, suggesting that, in parallel with embryogenesis, organogenesis also occurred in more differentiated tissue zones.

In contrast, the recalcitrant genotype PH16 exhibited a complete collapse of its developmental pathway. Its calli displayed predominantly large, vacuolated, non‐embryogenic cells with a whitish appearance (Figure 6d,e). The few cells with embryogenic potential and the formerly active meristematic centers regressed or remained restricted to small internal regions. This scenario confirms that the paradoxical and disorganized development observed at earlier stages resulted in an unproductive pathway, culminating in the total loss of embryogenic competence in direct contrast to the stable and sustained progression recorded in the responsive genotypes (Figure 6e).

### 3.6 Callose Deposition Throughout the Different Stages of Somatic Embryogenesis

Callose deposition was analyzed as an indicator of cellular isolation and tissue compartmentalization to determine whether its spatial and temporal patterns were associated with proembryo individualization and the contrasting embryogenic responses of the evaluated genotypes. The analysis revealed distinct temporal and spatial patterns among the genotypes, particularly during the initial and final developmental stages, providing complementary insight into their contrasting embryogenic responses.

**FIGURE 7:**
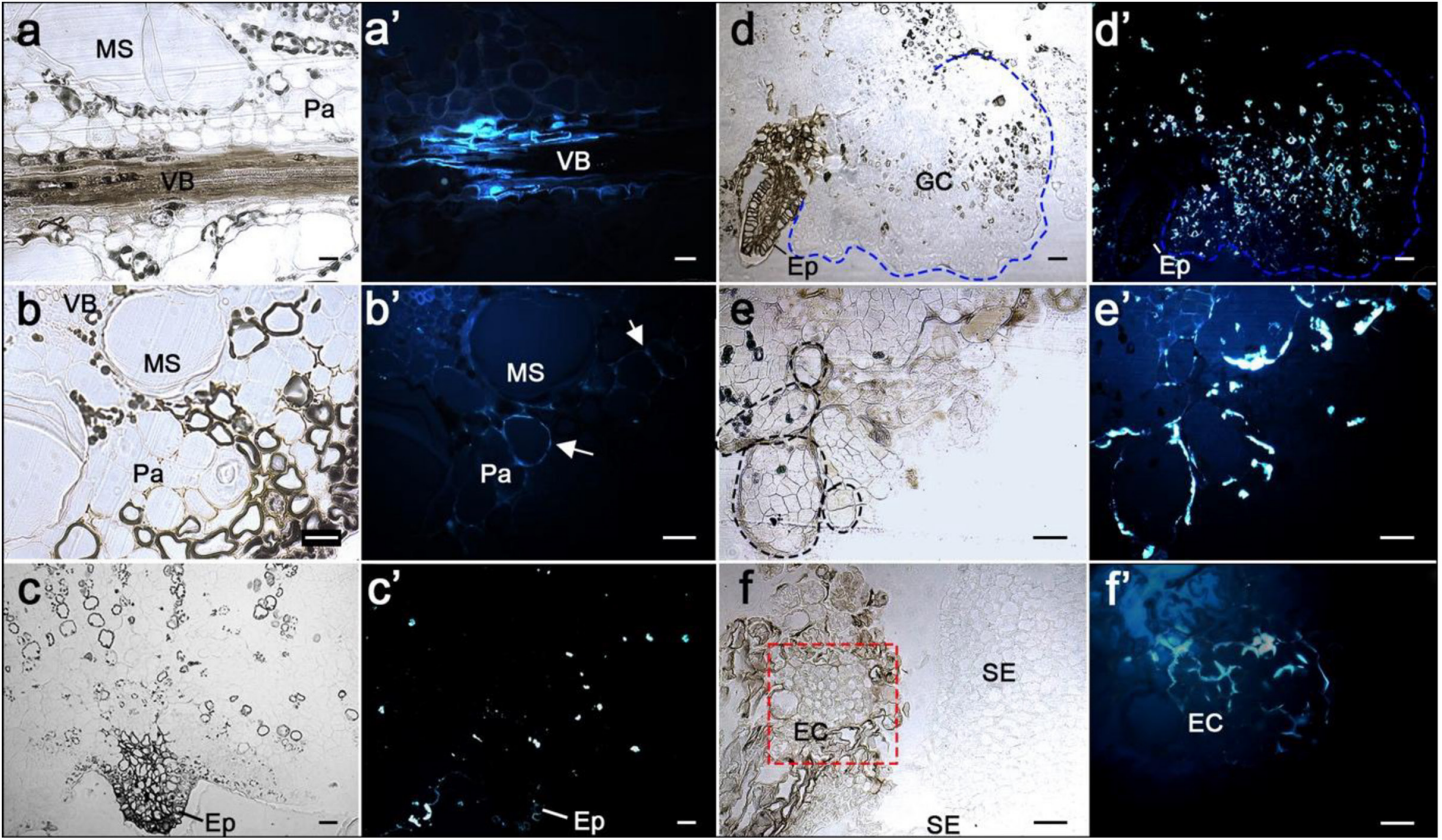
Patterns of callose deposition during somatic embryogenesis of *Theobroma cacao* . Temporal analysis (3 to 56 days) of callose deposition visualized by aniline blue fluorescence in explants of responsive genotypes (CCN‐51 and 752). Images (a–f) correspond to bright‐field microscopy and (a'–f') to callose fluorescence. Callose is sequentially observed in: (a, a') Vascular bundle cells (3 days); (b, b') Parenchyma cell walls adjacent to mucilage sacs (7 days); (c, c') Anatomically reactive areas of the parenchyma (14 days); (d, d') Dividing callus cells (21 days); (e, e') Embryogenic cells at the callus periphery and proembryos (42 days); (f, f') Deposition at the base of mature embryos and embryogenic masses (56 days). EC, embryogenic callus; Ep, epidermis; GC, granular callus; MS, mucilage sac; Pa, parenchyma; PEM, pro‐embryogenic mass; SE, somatic embryo; VB, vascular bundle. Symbols: White arrows indicates callose deposition; blue dotted line delineate granular callus; black dotted lines delineates proembryos. Scale bars: (a, a', b, b', f'): 20 μm; (c, c', d, d', e, e', f): 40 μm.

In the responsive genotypes, callose deposition was late and organized. At 3 days, it was minimal and restricted to the vascular bundles (Figure 7a). From 7 days onward, it expanded to parenchyma cells (Figure 7b), becoming concentrated at 14 days in regions of intense cell division (Figure 7c). At 21 days, the strongest signal was observed in expanding granular calli (Figure 7d). Between 28 and 42 days, callose formed barriers delimiting individualizing proembryos (Figure 7e). Finally, at 56 days, callose deposition supported the base of mature embryos, in the cells of the embryogenic callus, reinforcing continuous embryogenic competence (Figure 7f).

**FIGURE 8:**
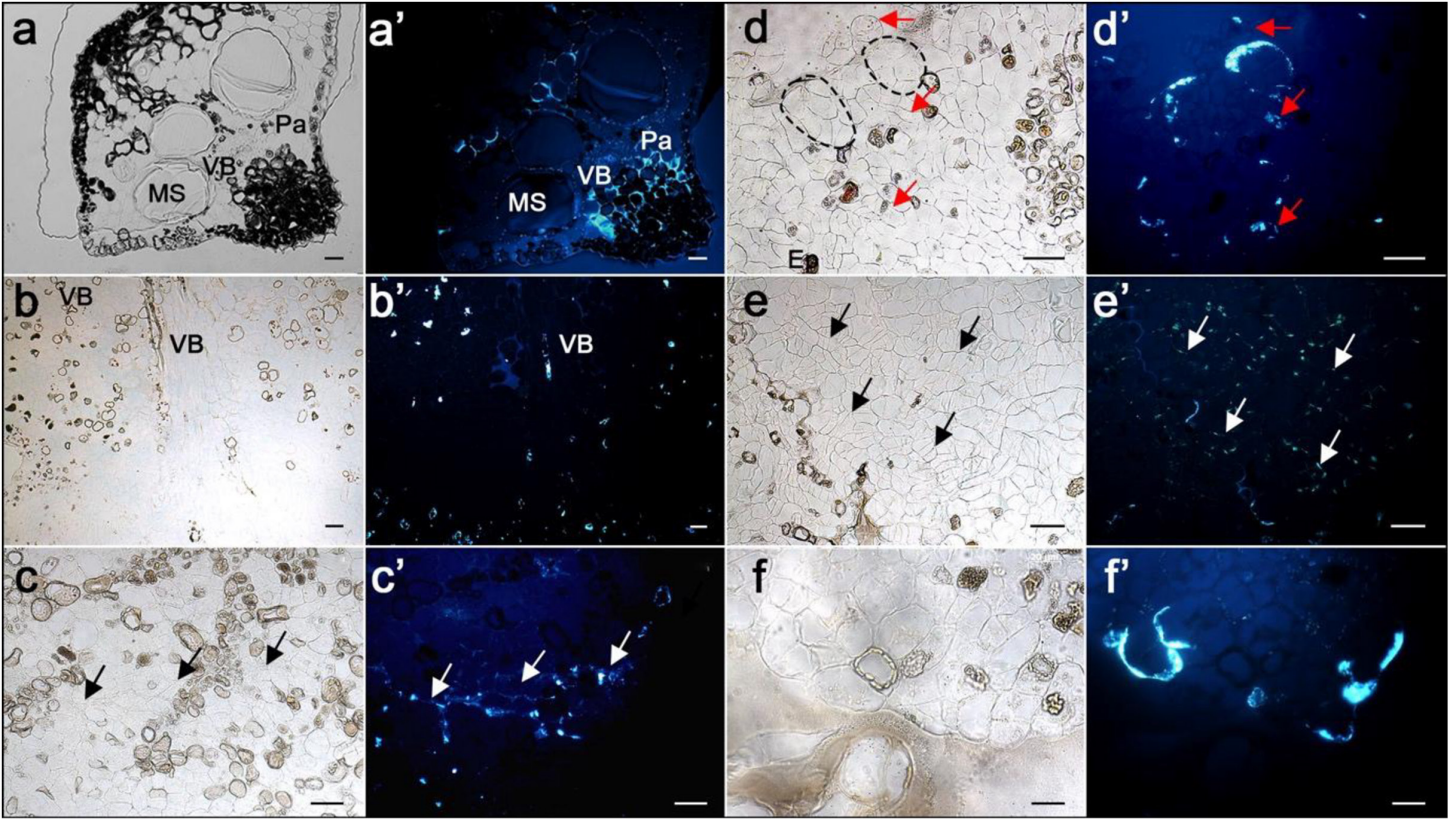
Patterns of callose deposition in explants of *Theobroma cacao* recalcitrant genotype PH16. Temporal analysis (three to 56 days) of callose deposition visualized by aniline blue fluorescence. Images (a–f) correspond to bright‐field microscopy and (a'–f') to callose fluorescence. Callose is sequentially observed in: (a, a') Deposition on the cell walls of the vascular bundle and parenchyma (3 days); (b, b') Accumulation in the cytoplasm and cell walls of parenchyma and vascular bundle cells (7 days); (c, c'): Deposition in dividing callus cells (14 days); (d, d') Signals in structures resembling proembryos at the callus periphery (21 days); (e, e') Weak and generalized deposition in cells showing disorganized proliferation (42 days); (f, f') Weak and diffuse deposition, especially at the callus edges and in the walls of recently divided cells (56 days). Ep, epidermis; MS, mucilage sac; Pa, parenchyma; VB, vascular bundle. Symbols: Black arrows show cell walls after cell division; black dotted lines indicate proembryos; white arrows show callose deposition on cell walls after cell division; red arrows indicate callose deposition in the cytoplasm. Scale bars: (a, a', b, b', c, c', d, d', e, e'): 40 μm; (f, f'): 20 μm.

In contrast, the recalcitrant genotype PH16 exhibited an early and diffuse response. As early as 3 days, callose deposits were visible in parenchyma cells (Figure 8a), suggesting a generalized stress response. Although present in dividing cells at 7 and 14 days (Figure 8b,c) and capable of delineating some structures at 21 days (Figure 8d), these deposits never reached the spatial precision observed in the responsive genotypes. In the later stages (28–56 days), deposition became weak and broadly distributed, occurring in disorganized proliferative cells and failing to establish the isolation barriers necessary for embryonic development (Figure 8e,f). Thus, the inability of PH16 to modulate callose deposition both spatially and temporally, progressing from an initial stress response to a precise structural function, may represent a key mechanism underlying its failure to sustain somatic embryogenesis.

### 3.7 Auxin Distribution by Immunolocalization at Different Stages of Somatic Embryogenesis

**FIGURE 9:**
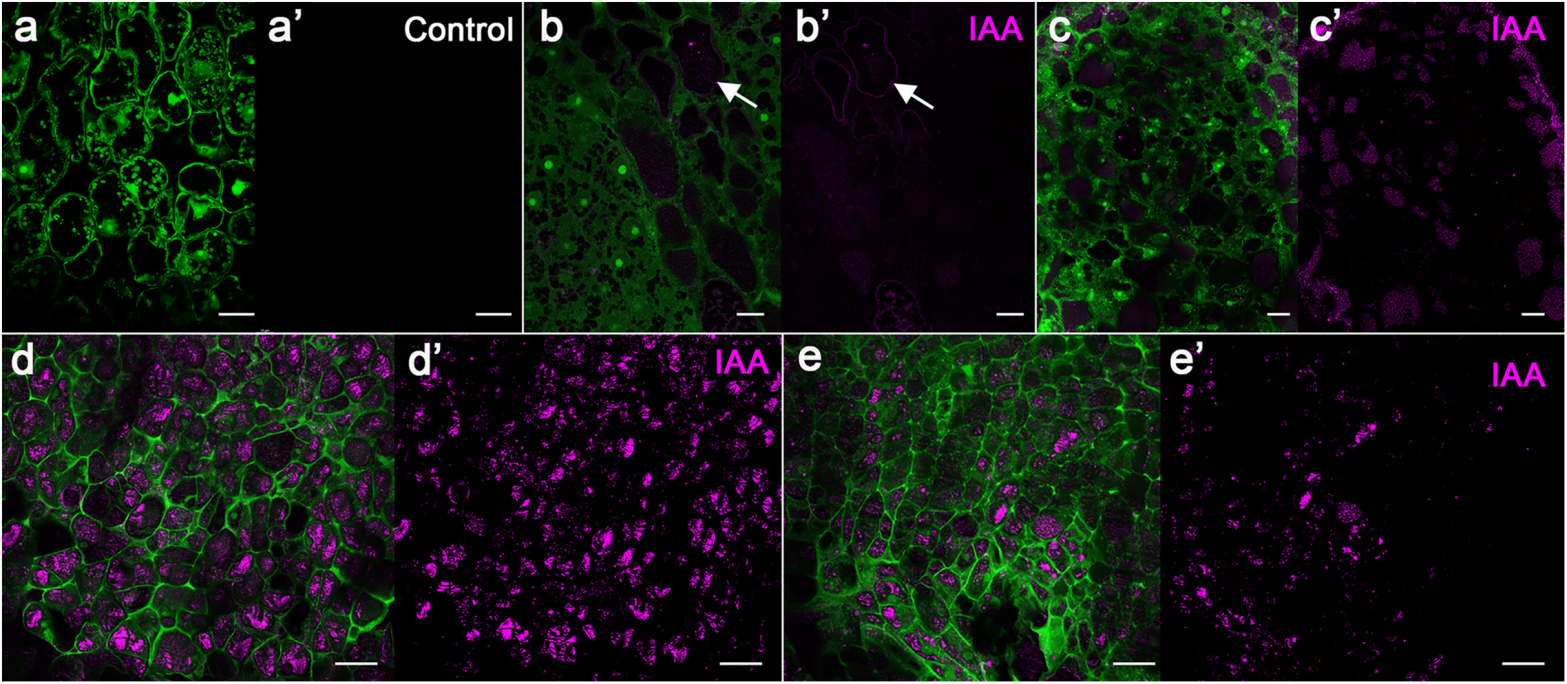
Immunolocalization of auxin during somatic embryogenesis in *Theobroma cacao* . Confocal microscopy images following immunolabeling with anti‐IAA primary antibody and Alexa Fluor 594‐conjugated secondary antibody. The immunofluorescence signal is shown in magenta, whereas tissue autofluorescence is shown in green, displaying auxin distribution across different stages of embryogenic development. (a, a') Negative control omitting the primary antibody. (b, b') 21 days: Discrete signal localized in the cell walls of cells near meristematic regions. (c, c') 28 days: Evident fluorescence in both cell walls and cytoplasm of cells in nodular calli. (d, d') 42 days: Maximum labeling intensity, with auxin accumulation in areas consistent with active meristematic centers. (e, e') 56 days: Signal still present but reduced, concentrated in regions of active cell division. Symbols: White arrows indicate discrete auxin signals localized in cell walls. Scale bars: (a, a'): 20 μm; (b, b', c, c'): 10 μm; (d, d', e, e'): 25 μm.

To investigate the hormonal dynamics associated with the embryogenic pathway, endogenous auxin distribution was evaluated by immunolocalization in staminode‐derived cultures of the responsive genotype 752 collected at 0, 3, 7, 14, 21, 28, 42, and 56 days after induction. Samples collected from 0 to 14 days showed no detectable IAA labeling and are therefore not shown in Figure 9. Representative images from 21, 28, 42, and 56 days are presented. Green autofluorescence resulting from the glutaraldehyde used during fixation provided morphological contrast for the cellular structures, whereas negative controls confirmed the absence of nonspecific labeling (Figure 9a).

Discrete signals appeared only at 21 days after induction, primarily located in the cell walls adjacent to developing meristematic centers (Figure 9b). By 28 days, labeling intensified and was distributed throughout the cytoplasm of cells within the nodular calli (Figure 9c).

Auxin accumulation reached its peak at 42 days, showing astrong and widespread signal in the most active meristematic centers, coinciding with the period of maximal organization of embryogenic callus and the emergence of the first somatic embryos (Figure 9d). Finally, at 56 days, the signal decreased but remained concentrated in areas of intense cell division. This reduction may reflect hormonal redistribution during the later developmental stages, occurring even after the removal of exogenous 2,4‐D from the culture medium (Figure 9e).

## 4 Discussion

The progression of SE in *T. cacao* demonstrates that embryogenic competence is less associated with the precocity of the cellular response and more with its stability and spatial organization over time. The recalcitrant genotype PH16 exhibited early and diffuse cellular reprogramming but was unable to sustain somatic embryo maturation. In contrast, the responsive genotypes 752 and CCN‐51 displayed a slower activation restricted to vascular tissues, yet with continuous and organized progression. The vascular origin of SE is consistent with the role of these tissues as reservoirs of stem cells or highly plastic cells essential for regeneration (De Rybel et al. 2016; Hunziker and Greb 2024).

Unlike the observations of Maximova et al. (2002), who reported somatic embryos only after 30–60 days, our results revealed relevant cellular events as early as 3–7 days after induction, providing new anatomical markers of responsiveness. Targeted activation of the embryogenic program within specific cellular niches, such as vascular tissues, appears to be a key determinant for successful somatic embryogenesis in responsive genotypes. This pattern, also reported in other species (Long et al. 2022; Silva‐Cardoso et al. 2022), reinforces the hypothesis that recalcitrance is associated with the inability to restrict induction to cells with true regenerative competence, resulting in a diffuse, disorganized, and ultimately inefficient response.

The accelerated development of the recalcitrant genotype PH16, which formed proembryos and even visible somatic embryos at 21 days, earlier than the responsive genotypes, reveals a central paradox: the speed of the response is not synonymous with success. On the contrary, this precocity should be interpreted with caution, as it suggests an imbalance in morphogenetic regulation. Unable to sustain its progression, PH16 demonstrated that a rapid and diffuse induction represents a disadvantage. The literature supports this interpretation, indicating that successful somatic embryogenesis requires a highly controlled process of cellular reprogramming (Garcia et al. 2022; Fehér 2023). This process initially involves the orderly release of epigenetic repression on key embryogenic developmental genes, followed by the establishment of localized auxin gradients that define the embryo's architecture (Fan et al. 2022; Long et al. 2022; Karami et al. 2023). Our results suggest that the massive and diffuse cellular induction observed in PH16 compromised the formation of these gradients, resulting in chaotic cell proliferation incapable of sustaining a structured embryogenic morphogenesis. Although PH16 exhibited clear limitations in progressing toward somatic embryo formation, definitive recalcitrance can only be asserted after systematic optimization of culture conditions, including medium composition, auxin type and exposure time, has been attempted and found ineffective. Therefore, under the conditions tested here, PH16 should be considered recalcitrant to somatic embryogenesis, rather than inherently non‐embryogenic.

Therefore, the apparently slow kinetics, spatially restricted to vascular tissues and observed in the responsive genotypes, should not be interpreted as a delay but rather as evidence of a more controlled and efficient developmental trajectory. This gradual pattern indicates the existence of a latency period required for the proper activation of the embryogenic program in pre‐existing stem cells, such as those of the procambium, rather than resulting from a disorganized dedifferentiation process (Silva‐Cardoso et al. 2022).

This process, in turn, depends on finely orchestrated epigenetic regulation, which is fundamental for the success of embryogenesis (Long et al. 2022; Li et al. 2025). Studies in *Hevea brasiliensis* support this view, that is, genotypes with high embryogenic capacity exhibit a dynamic DNA methylation pattern characterized by an initial demethylation phase that activates the embryogenic program, followed by a remethylation phase responsible for stabilizing the development. In contrast, recalcitrant genotypes show a continuous and deregulated increase in methylation, reflecting massive and disordered epigenetic control, as opposed to the precise and localized regulation observed in highly competent genotypes (Li et al. 2025). Future studies should map phase‐specific CG/CHG/CHH methylation in cacao via time‐course bisulfite sequencing and quantify global 5‐methylcytosine by HPLC to validate the proposed methylation trajectories. We suggest that the gradual response observed in the responsive genotypes enabled the activation of a key mechanism in embryonic development: the functional isolation of embryogenic structures through callose deposition. Our results, which show progressive callose accumulation around the proembryos, reinforce the role of this polysaccharide as a functional marker of embryogenic competence. Callose deposition promotes symplastic isolation by blocking plasmodesmata, an essential event that allows embryogenic cells to establish an autonomous developmental program. This mechanism is conserved across plants, as demonstrated in *Arabidopsis thaliana*, where callose deposition is a prerequisite both for the transition to the embryogenic fate and for proper embryo formation (Tao et al. 2012; Calabuig‐Serna, Mir, and Seguí‐Simarro 2023). In the present study, this pattern was also clearly observed in the genotypes 752 and CCN‐51, where callose deposits surrounded embryogenic structures at different stages between 28 and 56 days. In contrast, in the recalcitrant genotype PH16, the absence of organized isolation may have compromised embryo individualization and progression. Therefore, organized callose deposition not only restricts intercellular communication but also serves as a reliable marker of successful cellular reprogramming, consistent with observations in other species (Silva‐Cardoso et al. 2020; Hazubska‐Przybył et al. 2020).

In addition to structural isolation mechanisms such as callose deposition, the success of SE also depends on the precise coordination of intracellular signaling pathways. Calcium (Ca^2+^), recognized as a universal second messenger, plays a central role in this regulation (Pathak et al. 2020; Calabuig‐Serna, Mir, Arjona, and Seguí‐Simarro 2023; Calabuig‐Serna et al. 2025). Studies conducted in carrot, a model species for SE, have demonstrated that the formation of competent embryos is associated with the establishment of specific calcium gradients that are essential for defining embryonic polarity (Calabuig‐Serna, Mir, Arjona, and Seguí‐Simarro 2023). Although the present study did not directly assess such gradients, the disorganized cell proliferation observed in the recalcitrant genotype PH16 may hypothetically reflect the absence of efficient intracellular signaling, possibly including defects in calcium dynamics.

The dynamics of endogenous auxin provide a plausible framework for explaining the hormonal regulation underlying the distinct embryogenic responses observed among the evaluated genotypes. Because IAA distribution was evaluated only in the responsive genotype 752, the interpretations proposed for genotype PH16 should be regarded as hypotheses based on its contrasting morphoanatomical and developmental patterns.

In the recalcitrant genotype PH16, the failure to consolidate the embryogenic response suggests a possible inability to establish proper auxin gradients required for morphogenesis. This may also reflect limitations in the activation or maintenance of endogenous auxin biosynthesis, which is essential not only for triggering the embryogenic program but also for sustaining cell identity and promoting continuous embryo development. The diffuse cell proliferation observed in PH16 likely prevents the formation of localized auxin maxima, which depends on polar transport and is indispensable for maintaining meristematic niches and establishing morphogenetic patterns (Pasternak et al. 2002; Long et al. 2022; Silva‐Cardoso et al. 2022; Karami et al. 2023, 2024). Such disorganization may result from deregulated transcriptional control, as previously reported for genotypes with low embryogenic competence (Li et al. 2025). In contrast, in genotype 752, the only one for which auxin distribution was analyzed in this study, a late signaling peak was observed at 42 days, followed by a decline consistent with the embryonic maturation phase. This pattern of refined temporal regulation, consistent with previous findings (Hu et al. 2021; Silva‐Cardoso et al. 2022; Balcerowicz 2024), appears to be absent in PH16.

We suggest that failures in the regulation of the auxin transport machinery, under the conditions of this study, may underlie the limited response observed in PH16. One possibility is that this genotype exhibits an imbalance in efflux transport, preventing the proper intracellular accumulation of the hormone required to activate morphogenesis. This idea is consistent with studies showing that partial inhibition of auxin transport can, paradoxically, increase embryogenic efficiency (Balcerowicz 2024; Karami et al. 2024). Nevertheless, intracellular auxin accumulation depends not only on the transport but also on the endogenous synthesis, the deficiency of which may likewise compromise the maintenance of embryogenic identity and, consequently, proper embryo development (Carneros et al. 2023).

Alternatively, the dysfunction observed in PH16 may not be linked solely to the overall activity of auxin transport but also to the temporally and spatially misregulated expression of genes from the PIN‐FORMED (PIN) family, which are responsible for auxin efflux (Balcerowicz 2024). Successful somatic embryogenesis requires the coordinated activation of different PIN family members at specific stages of the process, such as the so‐called “Long PINs,” whose continuous expression is essential. A failure to activate the correct transporter at the appropriate time could interrupt embryonic development (Hu et al. 2021). Finally, deregulated interactions with cytokinins may antagonize PINs' expression (Pernisová et al. 2009; Bishopp et al. 2011), compromising the formation of the precise spatial auxin patterns that are prerequisites for successful embryogenesis.

The assembly of this complex hormonal signaling machinery is, in turn, regulated by master transcription factors of embryogenesis such as LEAFY COTYLEDON 1 (LEC1), LEC2, BABY BOOM (BBM), and WUSCHEL (WUS), which act as triggers of embryogenic identity (Henao‐Ramírez and Urrea‐Trujillo 2020; Li et al. 2022; Long et al. 2022; Fehér 2023). It has been demonstrated, for instance, that the BBM factor induces localized auxin biosynthesis, creating hormonal self‐sufficiency that is crucial for maintaining the identity and growth of somatic embryos (Li et al. 2022). Although such mechanisms were not directly assessed in this study, we suggest that the disorganized response in PH16 may reflect deregulation of these master transcription factors, whose coordinated expression is essential for embryogenic stability (Li et al. 2022). Future investigations comparing the expression of BBM, LEC, and WUS between responsive and recalcitrant genotypes may help clarify this point.

The final stage of cultivation highlights the fundamental distinction between “response competence” and “regeneration competence” (Nic‐Can et al. 2015). While the responsive genotypes exhibited the latter, PH16 illustrated the former, showing an initial cellular response that did not translate into complete regeneration. This limitation appears to be associated with its response pattern, characterized by early, intense, yet disorganized proliferation. Such behavior compromises the sequence of finely regulated events that are crucial for morphogenesis, including the establishment of hormonal gradients and the functional isolation of embryogenic structures.

This behavior is consistent with the collapse of regulatory mechanisms such as localized auxin and calcium signaling (Fehér 2019; Li et al. 2022, 2025), as well as with the absence of organized callose deposition observed in the responsive genotypes. Although not directly assessed in this study, it is plausible that this condition may be further aggravated by additional factors, such as the excessive production of phenolic compounds or secondary metabolites with self‐inhibitory effects, as previously described in other recalcitrant species (Nic‐Can et al. 2015; Gallego Rúa et al. 2016).

Therefore, this study reinforces that the true marker of success in somatic embryogenesis of *Theobroma cacao* is not the speed of cell proliferation but rather its spatial and temporal modulation. Our findings highlight the need for integrative approaches that combine anatomical, physiological, and molecular insights to refine protocols and overcome recalcitrance, enabling advances in the clonal propagation of elite genotypes.

From an applied perspective, these findings suggest that early histological markers may be useful for predicting embryogenic responsiveness before somatic embryos become visible. Genotypes exhibiting a spatially restricted and organized cellular response may represent more promising candidates for clonal propagation, whereas highly proliferative genotypes may require further optimization of culture conditions to promote more controlled embryogenic development.

## 5 Conclusions

Embryogenic competence in cacao is defined by the precise orchestration of a developmental program rather than a simple induction event. Regenerative success was linked to the activation of an organized program in vascular tissues, accompanied by localized auxin accumulation, callose deposition consistent with symplastic isolation, and putative epigenetic regulation as suggested by prior studies. In contrast, recalcitrance manifested as impaired “response competence,” characterized by uncontrolled proliferation and morphological/hormonal patterns consistent with epigenetic deregulation reported in other species, pending direct validation in cacao. To overcome these limitations in elite genotypes, it is essential to deepen the investigation of the molecular mechanisms governing the embryogenic transition, including DNA methylation dynamics and the expression of key genes, by comparing recalcitrant and responsive genotypes throughout the ontogenetic process. These findings provide new perspectives on the regulatory mechanisms of somatic embryogenesis in woody species and may support more effective strategies for optimizing clonal propagation protocols and for assisted selection in cacao breeding programs.

## Author Contributions

The study was conceived and designed by Jonny E. Scherwinski‐Pereira. Material preparation, data collection and analysis were performed by Stefhania Alzate‐Lozano, Inaê Mariê de Araújo Silva Cardoso, and Jonny E. Scherwinski‐Pereira. Claudia Franca Barros contributed to the anatomical interpretation and confocal microscopy imaging. Uilson Vanderlei Lopes coordinated the collection and genetic identification of donor genotypes and provided the source plant material. All authors commented on previous versions of the manuscript. All authors read and approved the final manuscript.

## Funding

This study was partially funded by the Coordenação de Aperfeiçoamento de Pessoal de Nível Superior, Brasil (CAPES, Finance Code process no. 88887.843829/2023‐00), through a doctoral fellowship awarded to Stefhania Alzate Lozano. Financial support was also provided by the Brazilian Ministry of Agriculture and Livestock (Ministério da Agricultura e Pecuária—MAPA) (SAIC 22200.21/0079‐1). Additional support was provided by the National Council for Scientific and Technological Development (Conselho Nacional de Desenvolvimento Científico e Tecnológico—CNPq, grant no. 313439/2023‐0).

## Conflicts of Interest

The authors declare no conflicts of interest.

## Data Availability

The data that support the findings of this study are available on request from the corresponding author.
